# Molecular Sticker Model Stimulation on Silicon for a Maximum Clique Problem

**DOI:** 10.3390/ijms160613474

**Published:** 2015-06-12

**Authors:** Jianguo Ning, Yanmei Li, Wen Yu

**Affiliations:** 1 State Key Laboratory of Explosion Science and Technology, Beijing Institute of Technology, Beijing 100081, China; E-Mails: jgning@bit.edu.cn (J.N.); liymsmail@gmail.com (Y.L.); 2 Beijing Key Laboratory of Intelligent Telecommunication Software and Multimedia, Beijing University of Posts and Telecommunications, Beijing 100876, China

**Keywords:** molecular computing, stickers model, maximum clique problem, SOPC

## Abstract

Molecular computers (also called DNA computers), as an alternative to traditional electronic computers, are smaller in size but more energy efficient, and have massive parallel processing capacity. However, DNA computers may not outperform electronic computers owing to their higher error rates and some limitations of the biological laboratory. The stickers model, as a typical DNA-based computer, is computationally complete and universal, and can be viewed as a bit-vertically operating machine. This makes it attractive for silicon implementation. Inspired by the information processing method on the stickers computer, we propose a novel parallel computing model called DEM (DNA Electronic Computing Model) on System-on-a-Programmable-Chip (SOPC) architecture. Except for the significant difference in the computing medium—transistor chips rather than bio-molecules—the DEM works similarly to DNA computers in immense parallel information processing. Additionally, a plasma display panel (PDP) is used to show the change of solutions, and helps us directly see the distribution of assignments. The feasibility of the DEM is tested by applying it to compute a maximum clique problem (MCP) with eight vertices. Owing to the limited computing sources on SOPC architecture, the DEM could solve moderate-size problems in polynomial time.

## 1. Introduction

DNA computers make use of DNA strands as the physical substrate in which information is represented, and the information is mainly manipulated through a set of useful operations on DNA strands. Their prominent advantage is making use of DNA molecules with enormous genetic code memory, and the immense parallelism of biochemical reactions. The essential work for DNA computing ability was revealed by solving a nondeterministic polynomial (NP)-complete problem, a seven-vertex Hamiltonian path problem [1]. Since then, many DNA-based models have been proposed, such as the sticker model [2], splicing model [3,4,5], hairpin model [6], the plasmid model [7,8], self-assembly model [9,10,11], and so on. Most are parallel filtering models with a large combinatorial library of solutions for the problem in question. These models are computationally complete and universal, and many NP-complete problems can be solved within polynomial runtime and exponential spaces [12,13,14,15,16].

However, a DNA-based computer has some limitations in terms of convergence speed, adaptability, and effectiveness due to the tools of biochemical laboratory, molecules encoding, detection technology, *etc.* Chang [17,18,19,20,21,22,23] proposed a biological computer inspired by molecular computing and solved several complex problems. The sticker model, as with typical DNA-based computers, has simple data structures called memory complexes, which are composed of a single-stranded DNA molecule and its associated stickers. According to the principle of Watson–Crick complementarity, the associated stickers could be annealed to or removed from the related complexes to realize bit-vertical operations. Reference [24] described a stickers model for the maximum clique problem and its implementation using a field programmable gate array (FPGA) architecture.

Inspired by the method of processing parallel information processing in the sticker model, a fundamental theoretical parallel model was proposed in our manuscripts [25,26] where we reported experiments performed theoretically to solve Boolean satisfiability problems, the 0–1 Knapsack problem. In this paper, we realize the theoretical model and propose a novel parallel computing algorithm on System-on-a-Programmable-Chip (SOPC) architecture. Firstly, we introduce the sticker model. Then, we realize DNA Electronic Computing Model (DEM) on SOPC architecture. An experiment in solving a maximum clique problem shows the feasibility and the application value of the model.

## 2. Results

The DEM uses System-on-a-Programmable-Chip (SOPC) architecture in which custom intellectual property (IP) soft core is embedded based on FPGA. The scan-driving controller and address-driving controller are packaged as the IP component of SOPC Builder. PDP is regarded as an I/O external device, and the instruction controller is implemented in NiosII.

The experimental platform uses the CORE4E-6DF Cyclone IV FPGA development kit of Altera as the core board, in which FPGA main chip uses EP4CE6F17C8N of the Altera high price Cyclone IV FPGA series. The target screen is two electrode PDP. Based on the existing experiment platform, an 8-vertex maximum clique problem (MCP) problem is taken as an example.

### 2.1. The Procedure of Experiment

**Figure 1:**
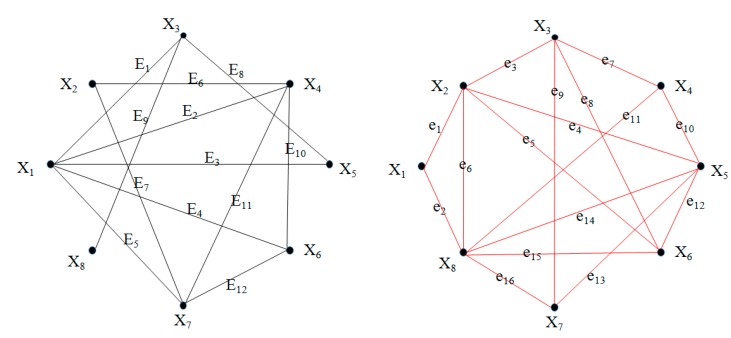
An undirected graph with 8 vertices and its complementary graph.

The undirected graph in Figure 1 has 8 vertices and 12 edges. The scan-driving controller for the graph has 8 input ports of *x*_11_, *x*_10_, *x*_21_, *x*_20_, x_31_, *x*_30_, *x*_41_, *x*_40_ and 16 output ports of *X_i_* in which *i* is from 0 to 15. The address-driving controller for the graph has 8 input ports of *x*_51_, *x*_50_, *x*_61_, *x*_60_, x_71_, *x*_70_, *x*_81_, *x*_80_ and 16 output ports of *Y_i_* in which *i* is from 0 to 15.

**Figure 2:**
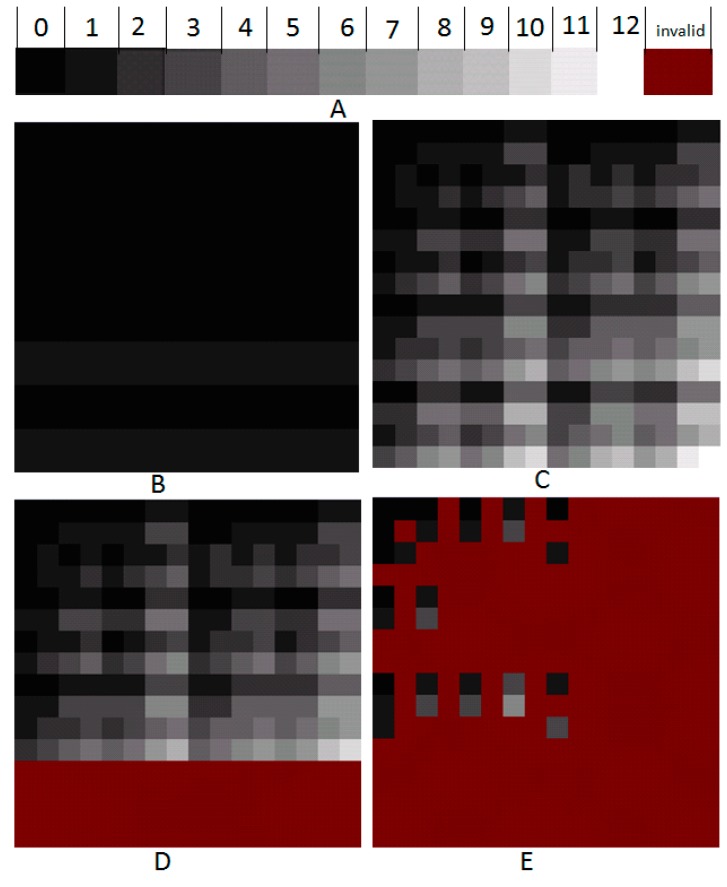
The procedure of the DNA Electronic Computing Model (DEM) for maximum clique problem (MCP). (**A**) Different grey levels for the numbers of edges and the last is the color of those invalid sub-graphs; (**B**) sub-graphs with edges *E*_1_; (**C**) all sub-graphs with edges from *E*_1_ to *E*_12_; (**D**) all sub-graphs within complementary graph; (**E**) all sub-graphs eliminated infeasible solutions.

Firstly, different grey scales are defined for the different numbers of edges and different colors of the sub-graphs Figure 2A. According to the Equations (5) and (6), the relationship between the input and output signals for scan-driving and address-driver controller are showed in Table 1.

Then, MCP solution space is obtained in 2*M* steps in which *M* is the number of edges in the graph. Take the edge *E_1_* as an example; the vertices are *x*_1_ and *x*_3_. Thus, the sub-graphs in which *x*_1_ = 1, *x*_3_ = 1 regardless of other variables, should all be set to be 1 in the first subfield. Otherwise, the left sub-graphs should be set to be 0 in this subfield. For the convenience of operation, all the sub-graphs are set to be 0 at first, and the related sub-graphs are set to be 1 in the first subfield. In the state of four-letter logic, *x*_1_ = 10, *x*_3_ = 10, and other variables are 11. Here, *x_i_*_1_*x_i_*_0_ is used to denote *x_i_*, so, *x*_11_= 1, *x*_10_ = 0, *x*_31_= 1, *x*_30_ = 0. According to Table 1, all addressing signals are 1, and the scanning signals of *X*_10_, *X*_11_, *X*_14_, *X*_15_ are 1. That is to say, the 10th, 11th, 14th, 15th lines are all set to be 1 in the first subfield. The result is displayed in Figure 2B. Assume that there are *M* edges in the graph, all the sub-graphs can be shown in different colors according to the number of edges in 2*M* steps. The image in Figure 2C depicts the result.

**Table 1:** The scanning and addressing signals of DEM for MPC with 8 vertices.

Scanning Signals	Value	Addressing Signals	Value
*X*_0_	$x_{10}\wedge x_{20}\wedge x_{30}\wedge x_{40}$	*Y*_0_	$x_{50}\wedge x_{60}\wedge x_{70}\wedge x_{80}$
*X*_1_	$x_{10}\wedge x_{20}\wedge x_{30}\wedge x_{41}$	*Y*_1_	$x_{50}\wedge x_{60}\wedge x_{70}\wedge x_{81}$
*X*_2_	$x_{10}\wedge x_{20}\wedge x_{31}\wedge x_{40}$	*Y*_2_	$x_{50}\wedge x_{60}\wedge x_{71}\wedge x_{80}$
*X*_3_	$x_{10}\wedge x_{20}\wedge x_{31}\wedge x_{41}$	*Y*_3_	$x_{50}\wedge x_{60}\wedge x_{71}\wedge x_{81}$
*X*_4_	$x_{10}\wedge x_{21}\wedge x_{30}\wedge x_{40}$	*Y*_4_	$x_{50}\wedge x_{61}\wedge x_{70}\wedge x_{80}$
*X*_5_	$x_{10}\wedge x_{21}\wedge x_{30}\wedge x_{41}$	*Y*_5_	$x_{50}\wedge x_{61}\wedge x_{70}\wedge x_{81}$
*X*_6_	$x_{10}\wedge x_{21}\wedge x_{31}\wedge x_{40}$	*Y*_6_	$x_{50}\wedge x_{61}\wedge x_{71}\wedge x_{80}$
*X*_7_	$x_{10}\wedge x_{21}\wedge x_{31}\wedge x_{41}$	*Y*_7_	$x_{50}\wedge x_{61}\wedge x_{71}\wedge x_{81}$
*X*_8_	$x_{11}\wedge x_{20}\wedge x_{30}\wedge x_{40}$	*Y*_8_	$x_{51}\wedge x_{60}\wedge x_{70}\wedge x_{80}$
*X*_9_	$x_{11}\wedge x_{20}\wedge x_{30}\wedge x_{41}$	*Y*_9_	$x_{51}\wedge x_{60}\wedge x_{70}\wedge x_{81}$
*X*_10_	$x_{11}\wedge x_{20}\wedge x_{31}\wedge x_{40}$	*Y*_10_	$x_{51}\wedge x_{60}\wedge x_{71}\wedge x_{80}$
*X*_11_	$x_{11}\wedge x_{20}\wedge x_{31}\wedge x_{41}$	*Y*_11_	$x_{51}\wedge x_{60}\wedge x_{71}\wedge x_{81}$
*X*_12_	$x_{11}\wedge x_{21}\wedge x_{30}\wedge x_{40}$	*Y*_12_	$x_{51}\wedge x_{61}\wedge x_{70}\wedge x_{80}$
*X*_13_	$x_{11}\wedge x_{21}\wedge x_{30}\wedge x_{41}$	*Y*_13_	$x_{51}\wedge x_{61}\wedge x_{70}\wedge x_{81}$
*X*_14_	$x_{11}\wedge x_{21}\wedge x_{31}\wedge x_{40}$	*Y*_14_	$x_{51}\wedge x_{61}\wedge x_{71}\wedge x_{80}$
*X*_15_	$x_{11}\wedge x_{21}\wedge x_{31}\wedge x_{41}$	*Y*_15_	$x_{51}\wedge x_{61}\wedge x_{71}\wedge x_{81}$

At the third step, all invalid sub-graphs are selected and set to 1 in the last subfield after steps.

Table 2 minutely shows the operation steps of MCP algorithm for Figure 1. Steps 1 to 12 are employed to generate an MCP solution space in which each step consists of two sub-steps, and steps 13 to 28 are used to eliminate all infeasible solutions.

**Table 2:** MCP operation steps of MCP algorithm for Figure 1.

Steps	Operation	Meaning
1	1.SW ******** 0	Sub-graphs with edges *E*_1_ are set to be 1 at 1th subfield
	2.SW 1*1***** 1	
2	1.SW ******** 0	Sub-graphs with edges *E*_2_ are set to be 1 at 2th subfield
	2.SW 1**1**** 1	
···	···	···
12	1.SW ******** 0	Sub-graphs with edges *E*_12_ are set to be 1 at 12th subfield
	2.SW *****11*1	
13	SW 11****** 0	Eliminate sub-graphs with edges *e*_1_ in the complementary graph at 13th subfield
14	SW 1******1 0	Eliminate sub-graphs with edges *e*_2_ in the complementary graph at 13th subfield
···	···	···
28	SW ******11 0	Eliminate sub-graphs with edges *e*_16_ in the complementary graph at 13th subfield

From Figure 2E, the maximum clique is the sub-graph with 6 edges in the 9th row and 6th column. According to the Equation (8), the addressing string can be got 10010110 by result-analyzing model. This means the maximum clique is {*x*_1_, *x*_4_, *x*_6_, *x*_7_}.

### 2.2. Time Complexity of the Algorithm

The operation time complexity of the above algorithm is the number of operations taken to solve the MCP. The algorithm includes four main steps. The first step has no operation. Step 2 consists of 2*M* operations. Step 3 consists of $C_{N}^{2}-M$, which is also the number of the edges of the complementary graph. Thus, the DEM can solve the problem in $C_{N}^{2}+M$ steps.

Not only is the operation time related to the number of vertices *M*, but also the number of edges *M*. Assume that edges density is β, then

(1) $$\text{β}=\frac{M}{C_{N}^{2}}$$

in which *M* is the number of edges, and $C_{N}^{2}$ is the maximum number of edges with *N* vertices. The operation time is $(1+\text{β})C_{N}^{2}=\frac{N\times (N-1)}{2}+M$. So the maximum clique problem for *G* can be solved in *o*(*n*^2^) operational time complexity.

**Figure 3:**
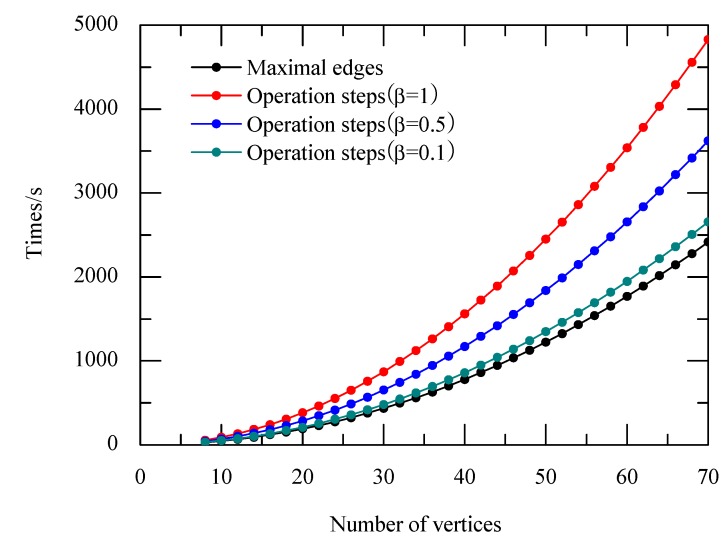
Time complex with density of edge.

Figure 3 shows that the runtime is polynomial in terms of the growing of vertex number. For the same number of vertices, the operation time increases from 0 at the smallest to 1 at the largest. In this way, the decisive advantage of the DEM is reflected distinctly. The exponential growth in time is shifted into space, and the runtime is polynomial.

### 2.3. Space Complexity of the Algorithm

Originally, in order to solve the maximum clique problem, the DEM need 2*^n^* SOPC memory to store the information and 2*^n^* PDP pixels to show the change of solutions, and n is the number of vertices. The main weakness of this library is that the number of memories required to represent all solutions will grow exponentially with the size of the problem. Consequently, it will generally be impossible to solve NP-complete problems using the DEM on SOPC architecture when the size of the problem is large.

Reference [10] introduced small combinatorial input libraries from which we can get some inspiration to deal the exponential growth of memories with the size of the problem. Reference [24] introduced a heuristic algorithm to address the limited FPGA memory. However, it is not guaranteed that the found cliques are maximal. Other methods will also be studied in the future research to overcome the demerit of the DEM.

### 2.4. Comparison with Previous Studies

So far, many methods have been proposed for solving the MCP. It is difficult to compare the DEM implemented on SOPC architecture with existing algorithm because the latter solvers are basically software methods run on different CPUs. However the operational time complexity could be used to compare their time efficiency.

Reference [27] introduced a sticker model based on FPGA architecture. The algorithm has a time complexity of *o*(*n*^2^) in solving k-clique, so it needs *o*(*n*^3^) time to solve the MCP.

Reference [28] presented an algorithm for MCPs based on DNA computation. The algorithm has a computation complexity of *o*(*n*^2^−|E|), where |E| is the number of edges in the graph.

In reference [29], the maximum clique problem for *G* can be solved using *o*(*n*^3^) operational time complexity, and *o*(*n*^3^) time complexity, where n is the number of edges of complement *G*.

In the DEM, the exponential growth in time is completely shifted into the spatial dimension. In this way, time efficiency is shown to be the outstanding advantage of the DEM over other methods.

## 3. DNA-Based Sticker Model

### 3.1. Representation of Information

It is generally accepted that the Watson–Crick complementarity principle plays an essential role in the massive parallel information processing in DNA-based computers. DNA molecule is composed of four nucleotides: adenine, cytosine, guanine, and thymine, or A, C, G, and T for short. These four nucleotides are always present in A–T and C–G pairs (the so-called Watson–Crick complementarity) in the annealing of two single-stranded oligonucleotides to form double stranded molecules. Mathematically, these four nucleotides suggest that DNA computers use a four-letter alphabet {*A*, *T*, *C*, *G*} to encode information.

**Figure 4:**
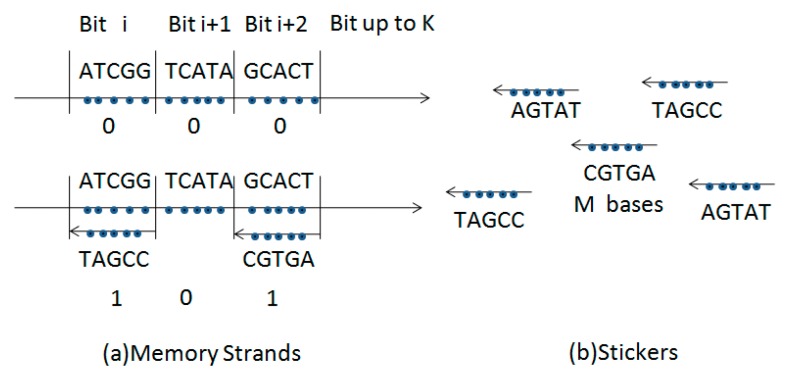
A memory strand and associated stickers (together called a memory complex) represent a bit string. (**a**) The top complex has all three bits off, the bottom complex has two annealed stickers and thus two bits on; (**b**) Associated stickers.

The typical sticker model [2] has a memory strand with *N* bases in length subdivided into *K* non-overlapping sub-regions each *M* bases long (thus *N* ≥ *MK*). The sub-regions, which are identified with exactly one bit position (or equivalently one Boolean variable), are significantly different from each other. Each sticker is *M* bases long and is complementary to one and only one of the *K* memory regions. If a sticker is annealed to its matching region on a given memory strand then a bit corresponding that particular region is on, or 1, for that strand. If no sticker is annealed to a region then that region’s bit is off, or 0. Indeed, memory strands are used as registers, and stickers are used to write and erase information in the registers in the sticker model. Figure 4 shows memory strands and associated stickers representing a bit string.

Another conception in the sticker model is (*K*, *L*) library. Each library contains memory complexes with *K* bit regions; the first *L* bit regions are either on or off, whereas the remaining *K* − *L* bit regions are off. The last *K* − *L* bit regions can be used for intermediate data storage. In every (*K* − *L*) library, there are at least 2*^L^* memory complexes.

### 3.2. Operations on Sets of Strings

A DNA-based computer is based on biochemical operation to realize computations. There are four main operations on sets of bit strings. The four principle operations are to combine two sets of strings into one new set, separate one set of strings into two new sets, and set or clear the *i^th^* bit of every string in a set.

## 4. DNA Electronic Computing Model (DEM) Method

### 4.1. Representation of Information

The DEM model employs the binary code of memory address and the content string to represent information. The memory address string is subdivided into *L* which is identified with exactly one Boolean variable during the course of computation, and each binary code bit can be labeled as a variable. These *L* variables (*i.e.*, the number of variables is *L*, *x*_1_, *x*_2_, ··· *x_L_*) can be used as the variables in NP-complete or other complex combinational problems. The content string of *K* length is also used for internal calculations.

Supposed that the binary code of *i* is *i*_1_, *i*_2_, ··· *i_L_*, then the value of variable *x_j_* is *x_j_* in which *j* is from 1 to *L*. For example, the binary code of the 5^th^ memory address string is 000101, so *x*_4_ and *x*_6_ are 1, and other variables are 0. When the variables from 1 to 6 are 000101 respectively, then the 5thmemory is selected. The memory address string labeled as *X_j_*, just as the literal meaning, is the address of memories and in some manners similar with the address signal in electronic computers.

**Figure 5:**
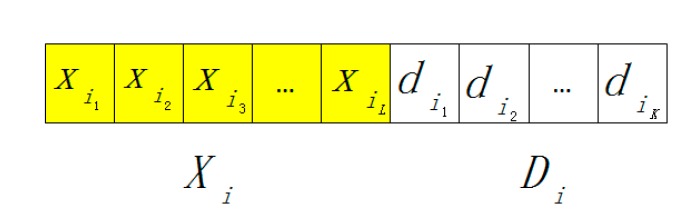
Information representation in DNA electronic computing model (DEM).

When *X_i_* is set to 1, the *i^th^* memory is selected and can be read or written. The *i^th^* content string is *D_i_* which can be denoted as *d*_1_, *d*_2_, ···, *d_K_*. The *i^th^* information representation string is displayed explicitly in Figure 5.

### 4.2. Processing of Information

To simulate the representation and parallel processing of massive information, the DEM uses a four-letter input alphabet *K* = {φ, 0, 1, *} to encode the memory address string. Note that the memory address string is based on the binary code 0 and 1; in other words, the output alphabet is 0 and 1. There is an inclusion relationship between input symbols:

(2) *⊃0,1⊃φ or (*⊇0,1⊇φ)

If the input address information string is “0*11”,it can be separated into the 3th (0011) and 7th (0111) strings according to the above relationship, that is to say, the “0011” and “0111” strings can be combined into “0*11”. Consequently, *X*_3_ and *X*_7_ are set to be 1, and the related memories are selected. The input address strings could be regarded as double stranded molecules in DNA computers, and the selected memory address string could be regarded as a single stranded molecule.

**Figure 6:**
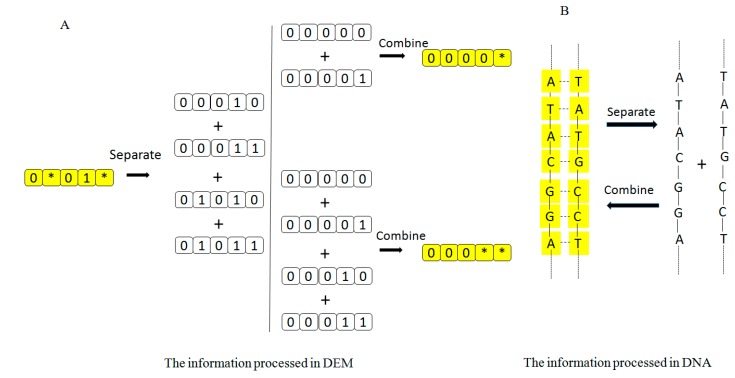
Processing of information in the DEM and DNA-based computer. (**A**) DEM can separate 0*01* into 00010, 00011, 01010, and 01011; DEM can combine 00000 and 00001 into 0000*; DEM can combine 00000, 00001, 00010, and 00011 into 0000**; (**B**) DNA-based computer can separate the left string into the right one, and combine the right one into the left one.

The processing of information in a DNA computer and in the DEM is compared in Figure 6. Figure 6A shows how information is processed in the DEM. The combine of the address information string is labeled with the yellow color, and the single address information string is in white. Figure 6B shows the processing of information in a DNA-based computer. The yellow components represent double stranded molecules. In the DEM, we can select all the 16 memories if we input “****”, while in a DNA computer, all single-stranded molecules can be also attached to all complementary molecules simultaneously in the process of annealing. Therefore, both approaches enable us to select as many memory addresses as possible to write or read at one step in parallel processing of massive amounts of information.

It should be noted that the input memory address string is of a four-letter alphabet, while the output memory address string is of 0 or 1. The *i^th^* memory could be selected only when the binary code of the memory address is included in the input string. There is a mapping relationship between the input memory address string of *L* length and all 2*^L^* decimal codes of memory address strings.

(3) $$\begin{array}{ll} F(V) & =X_{0}X_{1}...X_{2^{L}-1}\\ & =x_{0...00}x_{0...01}...x_{1...11} \end{array}$$

in which $V=v_{1}v_{2}...v_{L}\in K^{L}(v_{i}\in K)$ and *X_i_* (the binary representation is $x_{i_{1}}x_{i_{2}}...x_{i_{L}}$ represents the decimal code of the *i^th^* memory address.

(4) $$X_{i}=\left\{ \begin{array}{l} 1:\forall v_{k}⊇x_{i_{k}}(k=1,2,...,L),\\ 0:\text{otherwised.} \end{array}\right.$$

When *L* is 4, there are 16 memory address strings. If the input address string is *V* = 0*11, then the output memory address string is 0010001000000000. This means that the 3th and 7th memory logic units are activated.

Since electronic computers are based on two-letter logic (*i.e.*, 0 and 1), 00 is practically used to express φ, 01 is used to denote 0, 10 is used to denote 1, and 11 is used to denote *. The logical input address string “0*11” could thus be translated into 01111010.

### 4.3. Computation of DEM

DEM has two special states, SW and SR. SW is the state of simultaneous writing, and SR is the state of simultaneous reading. DEM can simultaneously write data into the selected memories in the state of SW at one step. DEM read data out from the selected memories in the state of SR. Data is * if the contents of the selected memories contain 0 and 1, 0 or 1 if the contents only contain 0 or 1.

Computation is implemented in the DEM as follows:

Step 1: Finite memories are selected according to the mapping relationship.

Step 2: Manipulate (S, V, d, i).

If the operation is SW, then DEM write d∈0,1 into the *i^th^* bit of selected memories.

If the operation is SR, then scan DEM the selected memories, and read out the *i^th^* bit of the contents to d∈0,1.

### 4.4. Example of DEM Computing

A maximum clique problem (MCP) is used as a benchmark problem to illustrate the power of the operations defined above.

Let *G* = {*V*, *E*} be an undirected graph with *N* vertices and *M* edges, where *V* = {*v*_1_, *v*_2_, ···, *v_N_*} is the vertex set of *G*, and E⊆V×V is the edge set of *G*. A graph is complete if all its vertices are pair-wise adjacent. The maximum clique asks for clique of maximum cardinality. For instance, the graph in Figure 1 has exactly one maximum clique{*x*_1_, *x*_4_, *x*_6_, *x*_7_}. Finding the maximum cliques in a graph is a NP-complete problem. This problem is quite important because it appears in many real world problems. Many important intractable problems turn out to be reducible to MCP—for example, the Boolean satisfiability problem and the vertex covering problem. Obviously, any two vertices connected in the original graph are not connected in the complementary graph, so the two vertices connected in the complementary graph cannot be 1 in the original graph at the same time.

The DEM algorithm for MCP is as follows:

Step 1: Define the information representation of the graph.

Step 2: Generate a solution space of (*N*, *M*) set, in which *N*is the number of vertices and *M* is the number of edges.

Step 3: Eliminate infeasible solutions.

Step 4: Find the maximum clique.

The first step is realized by constructing the information representation of the complete 2*^N^* solutions for a given graph with *N* vertices. All cliques are denoted as a set of binary numbers consisting of 0 and 1, in which 1 stands for the vertices in the cliques, and 0 represents the vertices out of the cliques. For instance, the maximum clique {*x*_1_, *x*_4_, *x*_6_, *x*_7_} is shown as 10010110. We utilize the binary code of memory address string as the set of 2*^N^* binary numbers.

In order to solve the MCP, we use content strings to denote *E*_1_*E*_2_···*E_M_* which has *M* bits in the set to indicate whether the edge is included in the clique. The content string of the clique {*x*_1_, *x*_4_, *x*_6_, *x*_7_} can be shown as 010110000111 because the clique has the edges *E*_2_, *E*_4_, *E*_5_, *E*_10_, *E*_11_, *E*_12_. The algorithm yields the following types of information representation: *X*_1_*X*_2_···*X*_8_|*E*_1_*E*_2_···*E*_12_. Thus, the clique {*x*_1_, *x*_4_, *x*_6_, *x*_7_} can be denoted as 10010110010110000111.

At the second step, we need to obtain all the solutions in which the binary address codes demonstrate the vertices and the binary content codes demonstrate the edges. In order to construct the binary content strings for every binary address string according to sub-graphs, we firstly initialize the *i^th^* bit of the content string to 0. The *i^th^* bit of the content string is 1 only when the two vertices *x_i_*_1_ and *x_i_*_2_ of the edge *E_i_* are both 1, so we can set these relative edges to be 1 at one step when the related memory addresses are selected.

At the third step, cliques of which vertices *x_i_*_1_ and*x_i_*_2_ are not connected, specifically connected in the complementary graph, are invalid. The following procedure is designed to eliminate the invalid cliques in which *i* is from 1 to $C_{N}^{2}-M$ *x_i_*_1_, *x_i_*_2_ are 1, and $C_{N}^{2}=\frac{N\times (N-1)}{2}$ is the maximum number of edges of graph *G* with *N* vertices.

After the above three steps, the maximum cliques are the sub-graph with the highest number of edges when there are still sub-graphs. The maximum clique is the binary code of the memory address string with the most number of 1in the content strings.

## 5. Architecture of DEM

**Figure 7:**
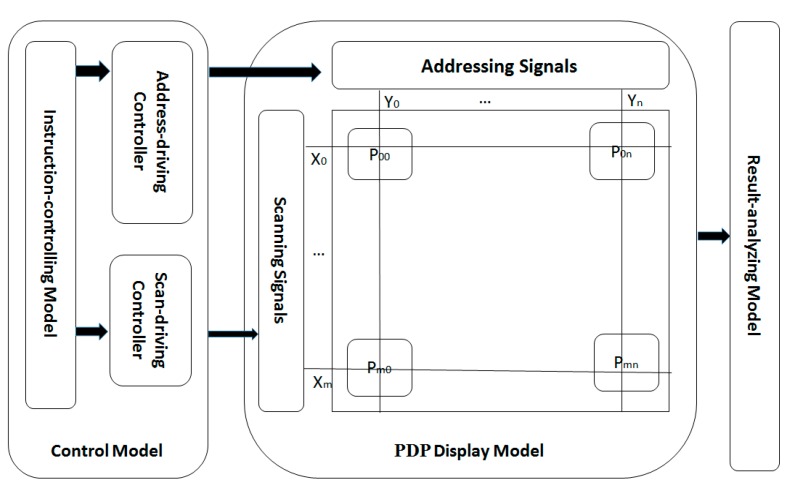
The functional architecture of DEM.

DEM is made up of three main parts from the functional aspect in Figure 7: control model, plasma display panel (PDP) display model, and result-analyzing model.

### 5.1. Plasma Display Panel (PDP) Display Model

At present, finding the true solutions from solution space for bio-molecular computing, namely the detection of a solution, is a complex issue. How to detect accurate solutions for problems in polynomial time is an important issue in eliminating the restriction on the development of DNA-based computers. Taking an NP complete problem as an example, if we could directly show the distribution of false solutions, truth solutions, or optimum solutions of the problem in different colors, we can solve the detection of solution easily.

PDP display model can scan the whole display panel at one subfield according to the input data. All subfields constitute the final picture after scanning. The binary codes of the display pixel address are labeled as memory address strings to represent the solution space, and the contents of display pixels are labeled as content strings to represent the edges in MCP.

Through progressive scanning, the address display period-separated subfield method (ADS) can simultaneously display image data in different grey scales in one field. Every edge can be seen as a subfield, so there are *M* subfields according to the number of edges. The subfield can be scanned to show when the associated edge exits. The grey scale of pixel is the sum of the associated sub-graph. Then, we need to delete the sub-graphs whose vertices exit simultaneously in the complementary graph. Notice that, the selected sub-graphs are set to red theoretically in order to show the infeasible sub-graph in step 3. The lightest valid pixels are the maximum clique.

**Figure 8:**
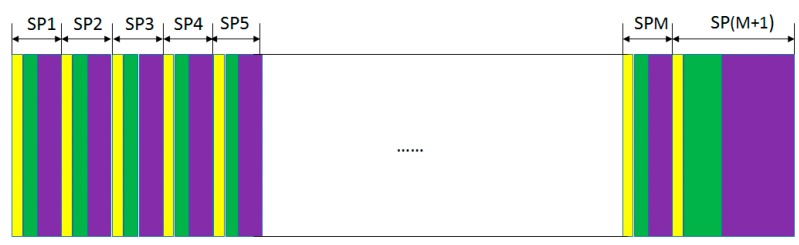
Address display period-separated subfield method (ADS) of DEM for maximum clique problem (MCP). The yellow colors mean the preparation period, the green colors mean the address period, and the purple colors mean the display period.

Figure 8 shows the address display period-separated subfield method of DEM for MCP. The subfields (SP) from 1 to *M* are operated respectively at one step. The subfield SP(*M*+1) is operated in $C_{N}^{2}-M$ steps.

When *N* is an even number, *N* = 2*n* (*n* = 1,2,3,4,···), the display resolution of PDP is 2*^n^* × 2*^n^*, that is, the number of pixels at each line is 2*^n^*, and there are 2*^n^* lines. When *N* is an odd number, *N* = 2*n* + 1 (*n* = 1,2,3,4,···), the display resolution of PDP is 2*^n^* × 2*^n^*^+1^,that is, the number of pixels at each line is 2*^n+^*^1^, and there are 2*^n^* lines.

### 5.2. Control Model

The Control Model is made up of three parts: the instruction-controlling model, address-driving controller (ADC), and scan-driving controller (SDC).

In order to become suitable for an implementation in SOPC, instructions are transformed into machine code. The full instructions and its machine code is illustrated in Table 3. The format of instruction is 4 bits of instruction followed by the pixel address of the display model and one operand.

Suppose that the input memory addressing strings are *X_i_* in which *i* is from 1 to *N*; in four-valued logic, *X_i_*_1_*X_i_*_0_ is used to denote the input address memory address *X_i_*.

**Table 3:** The instructions of the DEM.

Instructions	Machine Code	Meaning
(SR, V, d)	0001Vd	Read data from the selected memories
(SW, V, d)	0010Vd	Write data to the selected memories

#### 5.2.1. Scan-Driving Controller

**Figure 9:**
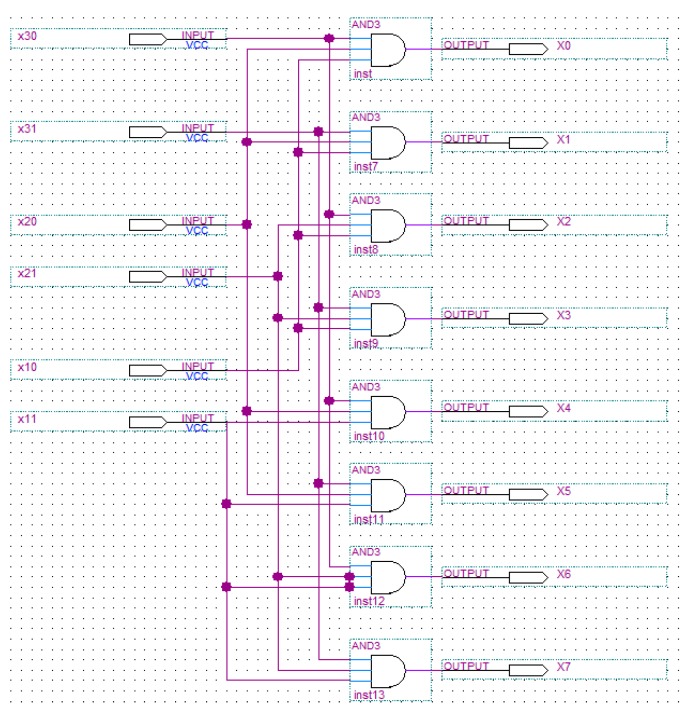
Scan-driving controller for *N* = 6.

There are 2*n* input ports and 2*^n^* output ports connected to scanning signals. The input signals are *x*_11_, *x*_10_, *x*_21_, *x*_20_, ···, *x_n_*_1_, *x_n_*_0_. If the binary code of *i* is *i*_1_*i*_2_*i*_3_···*i_n_*, in which *i* is between 0 and 2*^n^* − 1, then scanning signal *X_i_* is as follows:

(5) $$X_{i}=x_{1(i_{1})}\wedge x_{2(i_{2})}\wedge ...\wedge x_{n(i_{n})}$$

in which ^ means logic “and” operation. For example, if *N* is 6, there are 6 input ports, and 0 scanning signals. The binary code of 4 is 100, so $X_{4}=x_{1(1)}\wedge x_{2(0)}\wedge x_{3(0)}$. Figure 9 shows the circuit logic diagrams. Assume that *x*_1_ = 0, *x*_2_ = 0, *x*_3_ = * in four-letter logic (*i.e.*, *x*_11_ = 0, *x*_10_ = 1, *x*_21_ = 0, *x*_20_ = 1, *x*_31_ = 1, *x*_30_ = 1), the outputs *X*_0_ = 1, *X*_1_ = 1, and other outputs are 0 through the circuit. Thus, the first and second memory can be simultaneously selected to read or write in parallel.

#### 5.2.2. Address-Driving Controller

The input signals are *x*_(*n*+1)1_, *x*_(*n*+1)0_, *x*_(*n*+2)1_, *x*_(*n*+2)0_,···, *x_N_*_1_, *x_N_*_0_. When *N* is an even number *N* = 2*n* (*n* = 1,2,3,4,···), there are 2*n* input ports and 2*^n^* output ports connected to addressing signals. The binary code of *j* is *j*_1_*j*_2_*j*_3_···*j_n_* in which *j* is between 0 and2*^n^* − 1, then addressing signal *Y_j_* is as follows:

(6) $$Y_{j}=x_{\left(n+1)(j_{1}\right)}\wedge x_{\left(n+2)(j_{2}\right)}\wedge ...\wedge x_{\left(2n)(j_{n}\right)}$$

When *N* is an odd number *N* = 2*n* + 1 (*n* = 1,2,3,4,···), there are 2*n* + 1 input ports and 2*^n^*^+1^ output ports connected to addressing signals. The binary code of *j* is *j*_1_*j*_2_*j*_3_···*j_n_j_n+1_* in which *j* is between 0 and 2*^n^*^+1^− 1, then addressing signal *Y_j_* is as follows:

(7) $$Y_{j}=x_{\left(n+1)(j_{1}\right)}\wedge x_{\left(n+2)(j_{2}\right)}\wedge ...\wedge x_{\left(2n+1)(j_{n+1}\right)}$$

### 5.3. Result-Analyzing Model

The function of the result-analyzing model is to obtain all feasible assignments of MCP problem. Assume that the pixel in the *i^th^* row and *j^th^* column is the answer.

When *N* is an even number *N* = 2*n* (*n* = 1,2,3,4,···), binary code of *i* is *i*_1_*i*_2_*i*_3_···*i_n_*, the binary code of *j* is *j*_1_*j*_2_*j*_3_···*j_n_*, then the truth assignment is:

(8) $$i_{1}i_{2}i_{3}...i_{n}j_{1}j_{2}j_{3}...j_{n}$$

When *N* is an odd number *N* = 2*n* + 1 (*n* = 1,2,3,4,···) binary code of *i* is *i*_1_*i*_2_*i*_3_···*i_n_*, the binary code of *j* is *j*_1_*j*_2_*j*_3_···*j_n+1_*, then the truth assignment is:

(9) $$i_{1}i_{2}i_{3}...i_{n}j_{1}j_{2}j_{3}...j_{n}j_{n+1}$$

## 6. Conclusions

Inspired by the concept of information processing among bio-molecules, we propose a DNA Electronic Computing Model (DEM) that is a very novel combination of the advantages of traditional computers and DNA computing. Unlike previous molecular models, it does not rely on biotechnology, and requires no molecular strands or enzymes. The DEM has similar massive parallel operation characteristics with DNA computing power on a silicon-based computing medium.

The DEM seems well-suited to hardware implementation because of its deterministic faster operation. However, owing to the exponential growth of space with the size problem, it cannot be applied to large-scale data processing, and it is our future work to further improve the model for large-scale data processing.
